# *FAAH* rs324420 Polymorphism Is Associated with Performance in Elite Rink-Hockey Players

**DOI:** 10.3390/biology11071076

**Published:** 2022-07-20

**Authors:** Hugo-Henrique Silva, Valéria Tavares, Maria-Raquel G. Silva, Beatriz Vieira Neto, Fátima Cerqueira, Rui Medeiros

**Affiliations:** 1 ICBAS-Institute of Biomedical Sciences, University of Porto, 4050-313 Porto, Portugal; valeria.tavares@ipoporto.min-saude.pt; 2 Portuguese Ministry of Education, 1399-025 Lisbon, Portugal; 3 Senior Rink-Hockey Team, União Desportiva Oliveirense-Simoldes, 3720-256 Oliveira de Azemeis, Portugal; 4 Molecular Oncology and Viral Pathology Group, Research Center of IPO Porto (CI-IPOP)/RISE@CI-IPOP, Portuguese Oncology Institute of Porto (IPO Porto)/Porto Comprehensive Cancer Center (Porto.CCC), 4200-072 Porto, Portugal; i37300@ipoporto.min-saude.pt (B.V.N.); fatimaf@ufp.edu.pt (F.C.); 5 FMUP-Faculty of Medicine, University of Porto, 4200-072 Porto, Portugal; 6 FP-I3ID, FP-BHS, CEBIMED and Faculty of Health Sciences, University Fernando Pessoa, 4200-150 Porto, Portugal; 7 CIAS-Research Centre for Anthropology and Health—Human Biology, Health and Society, University of Coimbra, 3000-456 Coimbra, Portugal; 8 CHRC-Comprehensive Health Research Centre, Nova Medical School, Nova University of Lisbon, 1150-090 Lisbon, Portugal; 9 Scientific Committee of the Gymnastics Federation of Portugal, 1600-159 Lisbon, Portugal; 10 CIIMAR/CIMAR, Interdisciplinary Centre of Marine and Environmental Research, 4450-208 Matosinhos, Portugal; 11 Pathology and Laboratory Medicine Dep., Clinical Pathology SVIPO Porto Portuguese Oncology Institute of Porto, 4200-072 Porto, Portugal; 12 LPCC, Research Department, Portuguese League Against Cancer (LPPC—NRN), 4200-172 Porto, Portugal

**Keywords:** elite athlete, rink-hockey, gene, polymorphism, sport, performance, success

## Abstract

**Simple Summary:**

Genetic factors play a key role in athletes’ success; therefore, the interest for the study of the genetic profile contributing the most to extraordinary results in sport has been increasing. The aim of this study was to evaluate the impact of genetic determinants in the athletic performance of the world’s top best rink-hockey players. According to our results, elite rink-hockey players carrying the *Fatty Acid Amide Hydrolase* (*FAAH*) rs324420 A allele (AA or AC genotype) are three times more likely to be super athletes, which could be attributed to a higher pain tolerance and better stress coping, which may be useful for training and competition strategies. Despite the promising results, as this is a pioneer study, additional studies are needed for further validation of our results in rink-hockey and other sport modalities.

**Abstract:**

Genetic factors are among the major contributors to athletic performance. Although more than 150 genetic variants have been correlated with elite athlete status, genetic foundations of competition-facilitating behavior influencing elite performances are still scarce. This is the first study designed to examine the distribution of genetic determinants in the athletic performance of elite rink-hockey players. A total of 116 of the world’s top best rink-hockey players (28.2 ± 8.7 years old; more than 50% are cumulatively from the best four world teams and the best five Portuguese teams), who participated at the elite level in the National Rink-Hockey Championship in Portugal, were evaluated in anthropometric indicators/measurements, training conditions, sport experience and sport injuries history. Seven genetic polymorphisms were analyzed. Polymorphism genotyping was performed using the TaqMan^®^ Allelic Discrimination Methodology. Rink-hockey players demonstrated significantly different characteristics according to sex, namely anthropometrics, training habits, sports injuries and genetic variants, such as *Vitamin D Receptor* (*VDR*) rs731236 (*p* < 0.05). The *Fatty Acid Amide Hydrolase* (*FAAH*) rs324420 A allele was significantly associated with improved athletic performance (AA/AC vs. CC, OR = 2.80; 95% Cl, 1.23–6.35; *p* = 0.014; *p* = 0.008 after Bootstrap) and confirmed as an independent predictor among elite rink-hockey players (adjusted OR = 2.88; 95% Cl, 1.06–7.80; *p* = 0.038). Our results open an interesting link from FAAH-related biology to athletic performance.

## 1. Introduction

Elite athletes are thought to have an exceptional genetic potential [1] with incredible interindividual variability of physical performance traits (i.e., power, strength, endurance, muscle fiber composition and size, neuromuscular coordination and flexibility), personality profile [2], VO2 max (maximum oxygen consumption) and injury susceptibility [3].

According to Silva and coworkers [4], genetic factors play a key role in athletic success as they optimize gifted athletes’ achievements in comparison with less talented athletes. Additionally, the athletes’ training regime is also determinant in athletic accomplishments, i.e., the more intense the training, the better the results.

Remarkably, similar elite athletes in terms of physical characteristics can have distinct performance, with only few developing high-level performance abilities, making elite performance rare [4]. In fact, the decisive factor of outstanding performances is based on the athlete’s ability to perform not only optimal physical abilities but also emotional and mental competences [5].

The physical performance of athletes has been linked to more than 200 genetic variants, among which 155 correlate with elite athlete status [2]. In opposition, genetic foundations of competition-facilitating behavior and low stress response that influence elite performances are still scarce [4,5,6,7]. Additional investigation involving elite athletes is crucial to improve training and competition environments and provide more holistic treatment and recovery methods, especially in sports with a high risk of injury, as is the case of rink-hockey [8].

Rink-hockey is a high-intensity intermittent sport with noncontinuous movements at different speed levels and incomplete recovery periods [9,10]. Its effective playing time is 50 min, divided over two periods of 25 min (in categories of seniors, under 23 and under 19) [11], requiring regular endurance training to build tolerance for strenuous exercise and aerobic and anaerobic metabolisms (high-energy phosphate and glycolysis), as well as improving cardiovascular and musculoskeletal systems [9]. Furthermore, rink-hockey daily practice promotes morphological adaptations in the muscle spindle, which induce plastic changes in the central nervous system, decreasing the latency of the stretch reflex response and increasing its amplitude [12]. In particular, the player’s repetitive movements induce repetitive afferent inputs from the mechanoreceptors that over time promote plastic changes in the cortex [13], improving neuro-muscular efficiency, which is positively correlated with the sport competition level achieved [12].

In addition to these physical demands, this invasion game also requires a variety of mental abilities [14], particularly optimal decisions making, especially in the stressful last 15 min, when direct free hits, penalty shots and other one-against-one/two and three situations are decisive for the game final score.

Furthermore, given the high risk of potential injuries, mental abilities in the setting of rink-hockey are also required in order to cope with injuries and build pain tolerance [8,15]. In addition, rink-hockey goalkeepers are being increasingly considered of tremendous importance for teams’ decisive points, who should also be insightful in reading the game and resilience [16].

Therefore, psychological qualities such as planning skills in line with the game strategy, stress coping, anxiety management, resilience and avoiding impulsivity are determinant in athletic performance training and competition to achieve success [5]. Unfortunately, despite being a quite popular sport worldwide, there are few published studies on rink-hockey [15,17], among which very few are on genetics [4,18].

Previous studies have shown that genetic determinants among *Angiotensin-I-Converting Enzyme* (ACE), *Actinin Alpha 3* (ACTN3), *Angiotensinogen* (AGT), *NFE2-like BZIP Transcription Factor 2* (NRF-2), *PPARG Coactivator 1 Alpha* (PGC1A), *Peroxisome Proliferator-Activated Receptor Gamma* (PPARG) and *Transcription Factor A, Mitochondrial* (TFAM) genes are associated with sport-type (for instance endurance versus power) [2,5]. However, results are conflicted, and few genetic variants are linked to psychological traits and sport injuries [4] in relation to elite sport performance. Therefore, the present study aims to analyze genetic variants in genes coding for proteins potentially modulating the functioning of the brain’s center of emotions, located at the hypothalamic–pituitary–adrenal axis, especially those highly associated with the limbic system and their physiological pathways to adequately control a stress response [5]. For instance, the *Fatty Acid Amide Hydrolase* (FAAH) rs324420 is a polymorphism thought to play roles in neural functions and has been previously found to be associated with athletic performance in shaping anxiety-like behavior and affecting leadership and persistence [5,6]. To provide more insights into the topic, this study was designed to examine the impact of some of these genetic markers in a sample of elite rink-hockey players, particularly constituted by athletes competing in the Portuguese national championship (one of the world’s most competitive leagues, with the national team being the current world champion) [19], and test for any evidence of association with athletic performance.

## 2. Materials and Methods

### 2.1. Participants

Rink-hockey players who participated at the elite level (i.e., competing at any national team or other high-level representative teams in any sport organized by a National Sports Federation) [20], in the National Rink-Hockey Championship in Portugal during the sport seasons of 2019/2020, 2020/2021 and 2021/2022, were recruited to this study (*n* = 116). These athletes are the world’s top best rink-hockey players, as more than 85% of them are cumulatively from the best four world teams and the best five Portuguese teams, as recently published [21].

The participants were selected considering the following inclusion criteria: (1) being a minimum national-level athlete with an age equal or greater than 18 years old; (2) being an experienced competitor at the national level (i.e., a representative cohort of the world elite athletes competing in Portugal); and (3) having already reached a higher competitive level (i.e., international competitions, such as World, European or Continental Championships).

The athletes included in the study had rink-hockey experience for 23.3 ± 8.8 years and at the time of recruitment they were undergoing training programs of 5 to 6 days per week with an average of 2.4 ± 0.9 h per day, with a total of 13.8 ± 6.3 h/week. Additional characteristics of the participants are described in Table 1.

All athletes gave their informed consent to take part in this study and it was approved by the Ethical Committee of the University Fernando Pessoa (Porto, Portugal, Reference: CEUFP05062017).

### 2.2. Procedures

Data regarding anthropometric indicators/measurements, training conditions, sport experience and sport injuries history was collected using questionnaires personally administered to each participant before the training session by the same trained researcher.

#### 2.2.1. Anthropometric Profile

Body mass was measured by a digital scale (SECA-872, Hamburg, Germany) to the nearest 0.01 kg wearing T-shirt and gym shorts before training. Height was determined with a portable stadiometer (SECA-213, Hamburg, Germany) to the nearest 0.1 cm. Procedures were conducted as recommended by the International Society for the Advancement of Kinanthropometry [22]. Body mass index (BMI) was calculated as a ratio of weight to the squared height (kg/m^2^). Hand length was assessed with a flexible tape from the marked mid-stylion line to the most distal point of the third digit with the hand in a supinated position and fingers extended. The waist circumference (WC) was measured with a flexible tape at the end of a normal expiration at the smallest circumference between the thorax and the hips. The hip circumference (HC) was measured with a flexible tape at the largest circumference on trochanters and waist/hip ratio (WHR) was calculated.

#### 2.2.2. Training Data, Sport Experience and Sport Injuries History

Several parameters were obtained, namely the number of training sessions per week and the number of hours of training sessions per day, which were further used to calculate the number of hours of training per week. Sport experience included the athlete’s participations in regional and national teams, as well as national and international achievements. The elite athletic performance or success was defined according to the total number of national and international titles of the players. Based on tertiles, athletes were categorized as super athletes, i.e., if they had more than 11 titles (the highest tertile). Sport injury history was also collected (including the year of occurrence, anatomical part and time of recovery from first severe sport injury) [15].

#### 2.2.3. Sample Collection and DNA Extraction

Genomic deoxyribonucleic acid (DNA) was extracted from buccal cells samples of both sides (left and right) using sterile swabs (FL Medical, Hamburg, Germany), as previously described [23], through the use of the commercial kit QIAamp DNA Blood Mini Kit, Qiagen. Concentration and purity of DNA samples were assessed spectrophotometrically (NanoDrop Lite spectrophotometer, Thermo Scientific^®^, Waltham, MA, USA). DNA samples with a concentration equal to or higher than 4 ng/µL and with a purity (A260/A280) ranging between 1.70–2.00 were used for polymorphism genotyping.

#### 2.2.4. Polymorphism Selection and Genotyping

Recently, our research group conducted a review concerning all genetic factors associated with athletic performance across different sport modalities [4]. From the list of these factors (88 genetic variants), based on their previous association with athletic performance in a Caucasian population (endurance, power and/or sport injuries), the polymorphism minor allele frequency (MAF) in Iberian population (MAF should be higher than 10% to ensure genotype representation), their functional consequence, existence of validation studies and availability of TaqMan^®^ assays (Applied Biosystems), seven genetic polymorphisms were selected to be evaluated in our cohort of elite rink-hockey players (Table 2). Genotyping was performed in a Real-Time Polymerase Chain Reaction (RT-PCR) system (Applied Biosystems) using the TaqMan^®^ Allelic Discrimination methodology. All RT-PCR reactions were conducted in 6 μL volumes containing 2.5 μL of TaqMan^TM^ Genotyping Master Mix (2×), 2.375 μL of sterile water, 0.125 μL of 40× assay mix and lastly 1 μL of genomic DNA. Amplification conditions were the following: 95 °C for 10 min, followed by 45 cycles of 95 °C for 15 s and 60 °C for 1 min. Data capture and analysis were conducted on a Real-Time PCR System (Applied Biosystems) as described elsewhere [24]. To guarantee genotyping quality, two negative controls were included in each reaction, and double sampling was conducted in at least 10% of the samples randomly chosen, with an accuracy above 99%. The results were individually evaluated by two researchers with no previous knowledge regarding the athletes’ performance.

#### 2.2.5. Statistical Analysis

Statistical analysis was performed using SPSS software version 26.0 (SPSS, Inc., Chicago, IL, USA). Continuous data were expressed as mean ± standard deviation (SD), while categorical data were described in terms of frequencies. Some variables categories were defined based on median of variable values, namely: age (≥26 yrs. vs. <26 yrs.), WC/HC (≥0.85 vs. <0.85), hand length (≥23 cm vs. <23 cm) and time of recovery from first severe sport injury (≥10 months vs. <10 months). Associations between the genetic polymorphisms and athlete characteristics were assessed using student’s *t*-test for continuous variables, while chi-square test (χ^2^) or Fisher’s exact test were used for categorical ones. Using a binomial regression model, univariate analyses were conducted to identify predictors of elite athletic performance. Only the polymorphisms and other variables significantly associated with this trait in univariate analyses were further investigated in multivariate analysis to identify independent predictors. Bootstrapping analyses were performed through Monte Carlo simulation (1000 replications). Statistical significance was established at the *p* < 0.05.

## 3. Results

### 3.1. Characteristics of Elite Rink-Hockey Players

As expected, male rink-hockey players showed significantly higher anthropometric characteristics (body mass, height, BMI, WHR and hand length) than their female counterparts, who were mostly students (55.6%, Table 1). Since 45.9% of male athletes were professional rink-hockey players, they were also more experienced and submitted to a more intensive training program than their female counterparts (*p* < 0.05, Table 1) and suffered more sport injuries (*p* = 0.002, Table 1).

### 3.2. Genotype Frequencies in Elite Female and Male Rink-Hockey Players

The genotypes’ distribution of each polymorphism in female and male rink-hockey players is shown in Table 3. To be noted, the distribution of *Vitamin D Receptor* (*VDR*) rs731236 genotypes differed significantly between female and male athletes (*p* = 0.019) with the AG genotype being more prevalent among females (72.2% vs. 36.7%) and the AA and GG genotypes in males (45.9% vs. 22.2%, and 17.3% vs. 5.6%, respectively). Additionally, *Peroxisome Proliferator Activated Receptor Delta* (*PPARD*) rs2016520 CC, *Peroxisome Proliferator Activated Receptor Gamma* (*PPARG*) rs1801282 GG and the *Nitric Oxide Synthase 3* (*NOS3*) rs1799983 TT genotypes were absent among females. These results are most likely attributed to the reduced number of female athletes.

### 3.3. Univariate and Multivariate Analysis of FAAH rs324420 and Relevant Factors of Elite Performance in Rink-Hockey

According to univariate analysis, among the seven genetic polymorphisms, *FAAH* rs324420 was the only one significantly associated with athletic performance (AA/AC vs. CC; OR = 2.80; 95% Cl, 1.23–6.35; *p* = 0.014) (*p* = 0.008 after Bootstrapping analyses were performed through Monte Carlo simulation for 1000 replications). This effect was corroborated by multivariate analysis using a model with the factors age, sex, BMI, WC/HC, hand length, existence of severe sport injury and time of recovery from first sport injury (AA/AC vs. CC; adjusted OR = 2.88; 95% Cl, 1.06–7.80; *p* = 0.038; Table 4).

In this model, *FAAH* rs324420 and the existence of severe sport injury presented as independent predictors of elite athletic performance. According to the results, the rs324420 A allele seems to impose a benefit effect among elite athletes, as the carriers are three times more likely to be super athletes than those with CC genotype. As for the impact of severe sport injury (yes vs. no; adjusted OR = 3.71; 95% Cl, 1.21–11.31; *p* = 0.021; Table 4), it might be due to the high demands of athletic performance at this elite level.

## 4. Discussion

Most variants in genes coding for components of the cardiovascular, respiratory and musculoskeletal systems are thought to be associated with the elite athlete phenotype [2], but the input of genes coding for biochemical and structural elements of the brain regions associated with psychological traits have been less studied [5]. The aim of the present study was to examine the distribution of variants of genes with a potential role in nerve plasticity, neurotransmission, circadian rhythm, energy metabolism and antimicrobial and antitumoral activities in a sample of elite rink-hockey players and test for any evidence of association with elite athletic performance.

Our study group is unique as it comprises a high proportion of top, elite rink-hockey players, including those who achieved medals in the last World Championship.

In this study, as expected, significant differences between female and male athletes were observed, which can be explained by biological and professional-related factors. Inclusively, almost half of the male players were professional, whereas it was not the case for any female participant. As such, male athletes trained and competed more than their female counterparts, reporting also a higher incidence of sport-related injuries.

To the best of our knowledge, this is the first study evaluating the impact of genetic determinants in the athletic performance of elite rink-hockey players, as previous studies were mostly conducted on ice and field-hockey and at high school and college levels. Briefly, a study conducted with nine Spanish rink-hockey players with tendinopathy and five controls (rink-hockey players without tendinopathy) found that carrying one or more alleles A in *Gap Junction Alpha 1* (*GJA1)* rs11154027 (OR = 2.11; 95% CI, 1.07–4.19, *p* = 1.01 × 10^−6^) or G allele in *Vesicle Amine Transport 1-Like* (*VAT1L)* rs4362400 (OR = 1.98; 95% CI, 1.05–3.73, *p* = 9.6 × 10^−6^) was associated with a higher risk of tendinopathy [18]. On the other hand, carrying one or more A alleles in *Contactin-Associated Protein-Like 2 (CTNP2)* rs10263021 had a protective role (OR = 0.42; 95% CI, 0.20–0.91, *p* = 4.5 × 10^−6^). Sessa et al. [28] suggested a natural sports selection of an Italian group of athletes practicing intermittent sports (football, basketball and hockey players) whose frequency of Glu298Asp allele in *NOS3* rs1799983 affecting the blood pressure response to endurance training was higher compared with controls. A case-control study with Italian male and female elite athletes of basketball, soccer and hockey found that the polymorphism 5HTTLPR SS genotype of the *Solute Carrier Family 6 Member 4* (*SLC6A4)* was associated with neuroticism (*p* < 0.001) and adverse sport-related stress, namely tension/anxiety symptoms (*p* < 0.02), cognitive anxiety and emotional arousal control (*p* < 0.01) [7]. However, these two latter studies do not clearly indicate whether they included rink-hockey players (prevalent by sport and/or sex).

In fact, the little existing genetic research is mostly related to the other two disciplines of hockey (ice and field-hockey) and has been conducted in relation to sport performance (in ice-hockey [29,30,31,32] and in field-hockey [33]) and brain trauma in ice-hockey [34,35].

Even though it is consensual, the impact of sports injuries and/or traumas on the athlete’s physical and mental health and performance is devasting. The literature concerning the interaction between genetic and environmental factors and sports injuries is limited [36,37,38,39].

**Figure 1:**
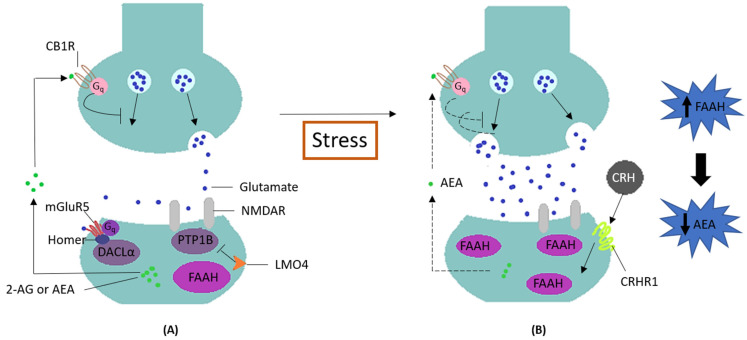
Endocannabinoid system as regulator of stress response. Under normal circumstances (**A**), the endocannabinoid system suppresses the release of the neurotransmitter glutamate via AEA, modulating the synaptic function. However, acute stress (**B**) can trigger a mechanism mediated by CRH and its receptor CRHR1, which is known to increase FAAH activity in the basolateral amygdala. As a result, there is a decrease in AEA levels, which is no longer able to suppress glutamate release. Consequently, increased neural excitability in the basolateral amygdala promotes anxiety-like behavior (adapted by Lutz et al.) [41]. CB1R: cannabinoid type 1 receptor; Gq: family G protein; mGluR5: metabotropic glutamate receptor 5; AEA: N-arachidonoylethanolamine; 2-AG: 2-arachidonoyl glycerol; DAGLα: diacylglycerol lipase-α; PTP1B: protein tyrosine phosphatase 1B; *FAAH*: *fatty acid amide hydrolase*; NMDAR: NMDA receptor; LMO4: LIM domain only 4; CRH: corticotropin-releasing hormone; CRHR1: corticotropin-releasing hormone receptor 1.

According to the study results, the incidence of severe sport injuries and *FAAH* rs324420 polymorphism are independent predictors of the athletic performance among our elite rink-hockey players, which may be due to significant differences according to the athletes’ sex and training frequency, as recently reported by Silva et al. [4]. As for rs324420, this missense SNP lies within *FAAH*, a gene encoding a protein (fatty acid amide hydrolase) reported as a promising target for the discovery of drugs to treat pain, inflammation and other pathologies [40], which is of great interest for athletic performance, in particular in a high-intensity and invasion sport as rink-hockey. This protein is a serine hydrolase engaged in the endocannabinoid metabolism, which degrades N-Arachidonoyl ethanolamide (Anandamide, AEA), a metabolite responsible for the activation of the cannabinoid type 1 receptor (CB1). Under stress exposure conditions (Figure 1), the protein FAAH is mobilized to degrade the AEA, therefore increasing the neuronal excitability in the amygdala, a key anxiety-mediating region of the brain [5]. In opposition, the inhibition of FAAH decreases anxiety-like behavior [41] and may produce an antidepressant effect mediated by CB1 receptor stimulation [42]. Previously, the rs324420 A allele was associated with a lower expression of FAAH levels [41]; in the population of the Iberian and African peninsula, it has a percentage of 16% and 37%, respectively [43]. Specifically, although the resultant protein displays normal catalytic properties, it shows an increased sensitivity to proteolytic degradation and shorter half-life, which explains the protein’ lower levels [5] (Figure 1). The SNP A allele might, therefore, be linked to a quicker habituation of amygdala reactivity to threat, decreased anxiety-like behavior, and increased fear extinction learning. This is of utmost importance for athletes to better cope with the personality trait of stress reactivity, deal more quickly with unpredictable situations and improve their motivation for sport competition [6,41,44]. This evidence is in line with our results, since the carriers of A allele (AA or AC genotype) were shown to be three times more likely to be super athletes than their counterparts (athletes with CC genotype), adjusted for other relevant factors (AA/AC vs. CC; adjusted OR = 2.88; 95% Cl, 1.06–7.80; *p* = 0.038).

In a previous report, Peplonska et al. [6] found that the polymorphism AA genotype is more common among sedentary controls than elite athletes (*p* = 0.0084), which led them to conclude that this genotype could have a negative impact on athletic performance. Similar findings were observed later [5]. In the study, which was conducted with 183 athletes of power, 212 of endurance sports and 451 controls, the authors observed that the SNP AA genotype was underrepresented in both power (in recessive model: OR = 0.36, 95% CI = 0.15–0.86, *p* = 0.017) and endurance athletes (in recessive model: OR = 0.42, 95% CI = 0.20–0.90, *p* = 0.022) compared with controls. In addition, this effect on athletic status was even more evident when the two groups of athletes were analyzed jointly (in recessive model: OR = 0.40, 95% CI = 0.22–0.72, *p* = 0.002), suggesting a negative impact on athletic performance [5].

Here, we hypothesized that such conflict results compared with ours might be attributed to the underrepresentation of AA genotype among elite athletes compared with sedentary controls, as our results suggest a benefit effect of the A allele in elite rink-hockey players when only elite athletes are considered. Additionally, rs324420 might play different roles depending on sport requirements, which needs to be further explored. Furthermore, although significant sex differences among our athletes were not observed for rs324420 genotype frequencies as previously described, it has been shown that estrogens modify emotional behavior through the dysregulation of the FAAH enzyme, thus causing an increase in the signaling of the endocannabinoid system, consequently decreasing anxiety in women [42]. Therefore, further studies would be needed to confirm this hypothesis in female athletes.

Overall, the observed effect of *FAAH* rs324420 is of utmost importance for future research in elite sport, since the mechanism of action of FAAH through the endocannabinoid system [45], applied to pain regulation and inflammation control, might improve mental and physical health and performance among elite athletes who face stressful environments daily [41].

As for the other six polymorphisms, no significant association was detected, despite the existence of some evidence linking these genetic variants to athletic performance [46]. Briefly, *PPARD* (implicated in fatty acid oxidation, cholesterol metabolism and thermogenesis) rs2016520 CC, *PPARGC1A* (involved in mitochondrial biogenesis, fatty acid oxidation, glucose utilization, thermogenesis, angiogenesis and muscle fiber type conversion toward slow-twitch type I fibers) rs8192678 TT genotype and rs8192678 G allele were associated with improved endurance performance (*p* < 0.01) [35,46,47,48,49]. The *PPARG* (implicated in adipocyte differentiation, glucose homeostasis and regulation of cardiovascular circadian rhythms) [50] rs1801282 CG genotype has been associated with decreased receptor activity and improved insulin sensitivity [51]. Regarding the *VDR* rs731236 variant, given the importance of vitamins in bone turnover, the polymorphism was associated with stress fractures (*p* < 0.05), with the GG genotype linked to higher multiple stress fractures when compared with their counterparts (*p* = 0.01) in a sample of 518 elite athletes (mean age 24.2 ± 5.5 years) of football, cricket, track and field, running events, rowing, boxing, tennis and others and controls [52]. The *Adrenoceptor Beta 2 (ADRB2*) (involved in the catecholamine system) rs1042713 A allele was associated with lower protein density (Beta-2 adrenoceptor), resting cardiac output and endurance performance [53]. On the other hand, the rs1042713 G allele was associated with sprint performance in elite youth football players [54]. As for *NOS3* (linked to several biological mechanisms, including angiogenesis) [50] rs1799983 polymorphism, this variant was previously associated with right ventricular structure and nitric oxide bioavailability in athletes’ cardiac adaptation. Its influence in the right ventricular structure was studied in elite water polo players, rowers, kayakers, canoeists and swimmers [53]. Although not significantly associated with sport performance, athletes carrying the rs1799983 T allele demonstrated increased right ventricular mass index (32 ± 6 g vs. 27 ± 6 g, *p* < 0.01) and larger right ventricular stroke volume index (71 ± 10 mL vs. 64 ± 10 mL, *p* < 0.01) compared with their counterparts [55].

Nevertheless, our findings concerning these polymorphisms need to be further validated given these previous results.

In terms of study limitations, the sample collection was highly affected by restrictions imposed by the coronavirus pandemic. The same condition also hampered the collection and analysis of some important factors, including for instance the percentage of adipose tissue and/or muscle mass, which can affect athletic performance.

Even so, the recognition of the study relevance by the study participants helped to minimize access constraints. Nevertheless, the participation rate is very representative of the elite in rink-hockey worldwide.

## 5. Conclusions

Elite rink-hockey players carrying the *FAAH* rs324420 A allele were three times more likely to be high-performance/super athletes, which could be attributed to a higher pain tolerance and better stress coping.

This is also of utmost importance for coaches, who can individually plan and adapt the athlete’s technical and tactical training and competition, keeping in mind the athlete’s specific ability to make decisions and support pain. Moreover, other sport professionals (e.g., medical doctors, physiotherapists, nutritionists and psychologists) should be aware of *FAAH* rs324420 and its associated consequences for the athlete’s increased resistance to pain and inflammation and time for injury recovery.

Despite the promising results, as this is a pioneer study, additional studies are needed for further validation of our results in rink-hockey and other sport modalities.

Furthermore, as few studies were conducted to access the role of *FAAH* rs324420, functional studies are also needed to better characterize this polymorphism and understand its impact on athletic performance. Given its thought biological impact, the role of this polymorphism should be explored in the setting of sport injuries among rink-hockey players, including type and frequency of injuries and recovery time.

## Figures and Tables

**Table 1:** Elite rink-hockey players’ characteristics (*n* = 116, 18 females and 98 males).

Characteristics	Female (*n* = 18)	Male (*n* = 98)	Total (*n* = 116)	*p*
Age (years) *	25.3 ± 7.9	28.8 ± 8.7	28.2 ± 8.7	0.117
Body mass (kg) *	67.4 ± 8.7	82.8 ± 10.5	79.0 ± 12.0	**0.000**
Height (m) *	1.73 ± 0.1	1.85 ± 0.1	1.82 ± 0.1	**0.000**
BMI (kg/m^2^) *	22.7 ± 2.6	24.3 ± 2.1	24.0 ± 2.2	**0.005**
WHR *	0.82 ± 0.06	0.88 ± 0.11	0.87 ± 0.11	**0.015**
Hand length (cm) *	22.3 ± 2.6	23.5 ± 2.0	23.3 ± 2.1	**0.029**
Training experience (years) *	19.4 ± 8.6	24.0 ± 8.7	23.3 ± 8.8	**0.039**
Training frequency *				
days/week	4.6 ± 0.8	5.9 ± 0.8	5.7 ± 0.9	**0.000**
hours/day	2.4 ± 0.9	2.4 ± 0.9	2.4 ± 0.9	**0.041**
hours/week	11.4 ± 6.5	14.3 ± 6.2	13.8 ± 6.3	**0.000**
Participations in regional and national teams *	26.44 ± 13.16	42.38 ± 37.78	39.9 ± 35.5	0.080
Sport injury				
No	17 (94.4)	51 (52.0)	68 (58.6)	**0.002**
Yes	1 (5.6)	47 (48.0)	48 (41.4)	
Nationality				
Portuguese	18 (100)	78 (79.6)	96 (82.8)	0.113
Others	--	20 (20.4)	20 (17.2)	
Ethnicity				
Caucasian	17 (94.4)	96 (98.0)	113 (97.4)	0.956
Others	1 (5.6)	3 (3.0)	3 (2.6)	
Profession				
Athletes	--	45 (45.9)	45 (38.8)	**<0.001**
Student	10 (55.6)	25 (25.5)	35 (30.2)	
Teacher	2 (11.1)	11 (11.2)	13 (11.2)	
Physical therapist	2 (11.1)	--	2 (1.7)	
Sport coordinator	--	1 (1.0)	1 (0.9)	
Coach	--	1 (1.0)	1 (0.9)	
Podiatrist	1 (5.6)	--	1 (0.9)	
Other	3 (16.7)	15 (15.3)	18 (15.5)	

* Data presented as mean ± standard deviation. BMI: body mass index, CI: confidence interval, OR: odds ratio, WHR: waist circumference/hip circumference ratio. Bold values represent significant results (*p* < 0.05).

**Table 2:** Selected polymorphisms’ description and TaqMan^®^ assays used.

Genetic Variant	Functional Consequence	Gene	Encode Protein and Its Functions	TaqMan^®^ Assays ID
			*Nerve plasticity and other neural functions:*	C___1897306_10
rs324420	*Missense*	*FAAH*	Fatty Acid Amide Hydrolase	
			*Energy metabolism and circadian rhythm:*	
rs8192678	*Missense*	*PPARGC1A*	PPARG Coactivator 1 Alpha	C___1643192_20
rs2016520	*5 prime UTR*	*PPARD*	Peroxisome Proliferator Activated Receptor Delta	C___8851952_30
rs1801282	*Missense*	*PPARG*	Peroxisome Proliferator Activated Receptor Gamma	C___1129864_10
rs731236	*Synonymous*	*VDR*	Vitamin D Receptor	C___2404008_10
			*Catecholaminergic system:*	
rs1042713	*Missense*	*ADRB2*	Adrenoceptor Beta 2	C___2084764_20
			*Neurotransmission, antimicrobial and antitumoral activities:*	
rs1799983	*Missense*	*NOS3*	Nitric Oxide Synthase 3	C___3219460_20

The functional consequence of each polymorphism was defined according to Ensembl database [25]. The functions of encoded proteins were described based on GeneCards database [26] and UniProt database [27].

**Table 3:** Genotype frequencies of the selected genetic polymorphisms in elite rink-hockey players (*n* = 116, 18 females and 98 males).

Genotype Frequencies	Female (*n* = 18)	Male (*n* = 98)	Total (*n* = 116)	*p*
***FAAH* rs324420**				
AA	1 (5.6)	3 (3.1)	4 (3.4)	0.789
AC	6 (33.3)	28 (28.6)	34 (29.3)	
CC	11 (61.1)	67 (68.4)	78 (67.2)	
***PPARGC1A* rs8192678**				
TT	2 (11.1)	17 (17.3)	19 (16.4)	0.705
CT	9 (50.0)	51 (52.0)	60 (51.7)	
CC	7 (38.9)	30 (30.6)	37 (31.9)	
***PPARD* rs2016520**				
CC	0 (0.0)	5 (5.1)	5 (4.3)	0.395
CT	7 (38.9)	32 (32.7)	39 (33.6)	
TT	11 (61.1))	61 (62.2)	72 (62.1)	
***PPARG* rs1801282**				
GG	0 (0.0)	4 (4.1)	4 (3.4)	0.494
CG	3 (16.7)	14 (14.3)	17 (14.7)	
CC	15 (83.3)	80 (81.6)	95 (81.9)	
***VDR* rs731236**				
GG	1 (5.6)	17 (17.3)	18 (15.5)	**0.019**
AG	13 (72.2)	36 (36.7)	49 (42.2)	
AA	4 (22.2)	45(45.9)	49 (42.2)	
***ADRB2* rs1042713**				
AA	3 (16.7)	15 (15.3)	18 (15.5)	0.989
AG	8 (44.4)	44 (44.9)	52 (44.8)	
GG	7 (38.9)	39 (39.8)	46 (39.7)	
***NOS3* rs1799983**				
TT	0 (0.0)	14 (14.3)	14 (12.1)	0.212
GT	11 (61.1)	47 (48.0)	58 (50.0)	
GG	7 (38.9)	37 (37.8)	44 (37.9)	

Bold values represent the significant results (*p* < 0.05).

**Table 4:** Multivariate analysis using binomial regression on the athletic performance considering *FAAH* rs324420 and relevant factors.

*Characteristics*	OR	95% CI	*p*
***FAAH* rs324420**	2.88	**1.06–7.80**	**0.038**
(*AA/AC* vs. *CC* ^1^)			
Age *	9.74	3.09–30.74	0.065
(≥26 yrs. vs. <26 yrs. ^1^)			
**Sex**	2.48	0.50–12.30	0.265
(*male* vs. *female* ^1^)			
**BMI**	0.58	0.19–1.80	0.349
(≥25 kg/m^2^ vs. <25 kg/m^2 1^)			
WHR *	0.83	0.30–2.28	0.721
(≥0.85 vs. <0.85 ^1^)			
**Hand length** *	0.35	0.12–1.02	0.054
(≥23 cm vs. <23 cm ^1^)			
**Sport injury**	3.71	**1.21–11.31**	**0.021**
(*Yes* vs. *no* ^1^)			
Recovery from first sport injury *	0.64	0.15–2.73	0.541
(≥10 months vs. <10 months ^1^)			

^1^ Reference group. * Variable categories were defined based on median of variable values. BMI: body mass index, CI confidence interval, OR odds ratio, WHR: waist circumference/hip circumference ratio. Bold values represent the significant results (*p* < 0.05).

## Data Availability

The authors confirm that the data supporting the findings of this study are available within the article.
